# The why, when, and how of computing in biology classrooms

**DOI:** 10.12688/f1000research.20873.2

**Published:** 2020-03-02

**Authors:** April M. Wright, Rachel S. Schwartz, Jamie R. Oaks, Catherine E. Newman, Sarah P. Flanagan

**Affiliations:** 1 Department of Biological Sciences, Southeastern Louisiana University, Hammond, LA, 70403, USA; 2 Department of Biological Sciences, University of Rhode Island, Kingston, RI, 02881, USA; 3 Department of Biological Sciences, Auburn University, Auburn, AL, 36849, USA; 4 Biology Program, University of Louisiana Monroe, Monroe, LA, 71209, USA; 5 School of Biological Sciences, University of Canterbury, Christchurch, 8042, New Zealand

**Keywords:** computation, biology, education, undergraduate

## Abstract

Many biologists are interested in teaching computing skills or using computing in the classroom, despite not being formally trained in these skills themselves. Thus biologists may find themselves researching how to teach these skills, and therefore many individuals are individually attempting to discover resources and methods to do so. Recent years have seen an expansion of new technologies to assist in delivering course content interactively. Educational research provides insights into how learners absorb and process information during interactive learning. In this review, we discuss the value of teaching foundational computing skills to biologists, and strategies and tools to do so. Additionally, we review the literature on teaching practices to support the development of these skills. We pay special attention to meeting the needs of diverse learners, and consider how different ways of delivering course content can be leveraged to provide a more inclusive classroom experience. Our goal is to enable biologists to teach computational skills and use computing in the classroom successfully.

## Introduction

Biology departments are increasingly offering or considering course work that incorporates hands-on computation ( Wilson Sayres *et al.,* 2018). There are different rationales for courses that include computation. Perhaps the most obvious rationale is to teach computational biology, bioinformatics, and/or skills in working with large datasets. Bioinformatic skills are increasingly a core component of biology and biology education, and support students’ job and/or graduate school preparation ( Wilson Sayres *et al.,* 2018). The vast majority of researchers in biology either use large datasets or could do so with sufficient training, suggesting that general training in effective use of large datasets is essential for students as well ( Barone *et al.,* 2017; Loman & Watson, 2013). However, even with small datasets, performing analyses reproducibly and having access to the wide range of statistical and analytical tools available in scientific computing languages improves the work of scientists. Thus, in coursework involving computation, instructors teach particular skills or tools that are an integral part of the field. An alternate motivation is to incorporate more data and analysis into course work. Integrating data into courses provides more opportunity for students to experience the research process, and the ability to incorporate active learning activities where students recognize biological theories by drawing conclusions from their own results ( Kjelvik & Schultheis, 2019). Another rationale for teaching computational skills is to increase data literacy ( Gibson & Mourad, 2018), which, in addition to enhancing learners’ preparation for future careers, also better prepares learners to think critically about societal issues relevant to science and policy ( Cook *et al.,* 2014).

However, there are significant barriers to teaching hands-on computation in biology classrooms ( Williams *et al.,* 2017). While many biologists use computation in their research, relatively few have explicit training in this area. Fewer still have training in how to teach computation ( Williams *et al.,* 2017). Importantly, few biologists are housed in a department with the existing expertise or infrastructure for teaching computation ( Williams *et al.,* 2017); thus, there is often less familiarity with options for teaching computing or recent best practices. Cummings & Temple (2010) describe the challenges in teaching bioinformatics as “infrastructure and logistics; instructor knowledge of bioinformatics and continuing education; and the breadth of bioinformatics, and the diversity of students and educational objectives.” Williams *et al.* (2017) similarly cites faculty training, infrastructure, and student interest and preparation. Here our goal is to address issues of infrastructure and logistics, and make recommendations for faculty practice. We suggest that faculty seeking computational training consider programs such as Software and Data Carpentry workshops as well as opportunities offered by QUBES, NEON, and their own universities. Additionally, we suggest that faculty separately seek training in teaching computation through programs such as the Software and Data Carpentry instructor training courses. This article explains options available for instructors to teach computational course content (collectively referred to here as computing platforms), discusses how to choose the best computing platform for a course, and describes effective strategies for teaching coding and data analysis.

An important consideration is that whichever technology an educator uses to deliver their content supports the educator’s learning objectives and enhances students’ ability to learn. Every course has a unique set of learning objectives, and every group of learners poses a unique set of challenges. Such learning objectives related to computing may range from engaging students in the value of basic coding skills to building advanced skills and the ability to self-teach and work independently. An example of the former might include a freshman introductory biology lab focusing on data analysis, where students focus on the results from the data analysis. In contrast, an upper-division course on research methods may focus more on preparing students for independent data analysis by emphasizing self-sufficiency in coding, reproducibility of the research, and software management and installation. Additionally, the selected platform needs to support the type of work the students are doing. In the first example, students might need to learn to make appropriate plots to visualize biological data; thus, the platform students work with needs to have the capacity to display plots. On the other hand, if a course objective is for students to learn to manage genome-scale data on a remote server, a platform using a command-line interface to access a computing cluster may be more appropriate.

Much as a biology laboratory course requires specific laboratory equipment, teaching computation requires learners to be able to access specific software and functions. This may range from one or a few pieces of software that can be downloaded from the internet to installation of a computing language and development environment. For simplicity, the interfaces used by learners and the instructor should be uniform, as different versions of software could have different interfaces or commands. Learners also need a way to compute outside of class — for example, to do the homework outside class hours. Finally, the computing platform should allow students to focus on the coursework and instruction. The learning platform must not interfere with students’ focus on the learning objectives.

Another consideration is the consistency and scaffolding in selecting tools and/or the larger platform. Gibson & Mourad (2018) cites the need for “coordinated, sequential development of data literacy across the biology curriculum”, which suggests the value of a platform and tools on which students can scaffold their skills, rather than each course choosing its own tool. For example, students would likely find it challenging to use R in one class, Python in another, and a genomics platform such as Galaxy in another ( Wu & Anderson, 1990). Furthermore, switching languages and platforms (i.e. learning a new skill set) can detract from time spent on other educational activities when the goal is not to explicitly teach the language or platform.

Every choice of computing platform comes with costs and benefits. In this article, we will discuss tools and techniques to facilitate teaching computation in courses with some degree of programming or data analysis. We explain different computing platforms with a special eye towards serving diverse sets of learners, in terms of their motivations, life experiences, and access to technology. In particular, we will focus on programming languages that are common in biology: R and Python. Lastly, we review effective pedagogical techniques for helping learners thrive in a computational classroom.

## Choice of computing language

One of the first choices an instructor needs to make when they plan coursework is what computing language to use. In biology, R and Python are common languages. Bash, a type of UNIX command-line language, is commonly used to run software and automate tasks. There are multiple considerations to take into account for this choice, including *consistency*, *current status of research tools*, and *comfort*. *Consistency* refers to consistency of the language’s use throughout the curriculum. Switching languages can be associated with learning loss in novices ( Wu & Anderson, 1990). Therefore, in order to deepen and strengthen learners’ command of languages, integrating the same language consistently throughout the curriculum may be helpful. For example, if an instructor is integrating a computational component into a genetics lab, they might check with a colleague teaching biostatistics or bioinformatics to see what language they are using.

The *current status of research tools* is also worth considering. If the goal of a course is for learners to understand both biology and computation, the instructor will want to choose a language with active development of tools for research in that field. For example, many genomics and phylogenetics tools are written in Python. On the other hand, more ecological and comparative biology tools are written in R. Therefore, the choice of language will inform the subset of tools available to the instructor and the learners.

Finally, instructors should consider their own *comfort*. Teaching requires fluent and comfortable discussion of concepts and tools. Instructor knowledge must factor into this. Additionally, community resources exist for some languages. For example, QUBES is a National Science Foundation-sponsored (NSF) project that publishes lessons and sponsors instructor mentoring groups in various scientific disciplines. An instructor may feel more empowered to teach well if there is a strong sense of community in their language, with pathways to ask for help and access resources.

It is also worth noting that not all tasks that are computational necessitate the addition of programming into the curriculum. For example, tools such as the NSF-sponsored Galaxy project allow instructors to help learners run bioinformatics and genomics software via a point-and-click internet interface. If the goal of a course is to demonstrate answering a particular question, but not to dig too deep into the methodology of how that is done, this type of interface may be sufficient. Teaching with these resources is beyond the scope of this article, as the Galaxy website provides extensive resources for teaching, and trainers provide regular workshops on this tool.

The R language allows inexperienced learners to immediately work with rectangular (i.e. spreadsheet-style) data in a way that is engaging. For example, a learner can load data and plot results within an hour of first opening RStudio. This engagement is important for learners who are more interested in effectively working with data than learning to code. We suggest that the R language immediately supports coding-to-learn with minimal learning-to-code. In particular, the tidyverse set of packages provided by RStudio provides convenient, well-supported, and intuitive functions for plotting and manipulating data. Bioconductor ( Huber *et al.,* 2015) has a wide variety of packages related to genomic data, as well as tutorials for this use, while other packages such as ape ( Paradis & Schliep, 2018) provide resources for specific tasks such as reading and viewing phylogenies, and many other packages are available with easy-to-follow vignettes.

Python is a general purpose programming language used in a wide variety of industries and can be adapted to many purposes. For example, the popular data science library Pandas ( McKinney, 2010) can be used to teach foundational data skills such as managing rectangular data. Python also has a variety of resources for teaching work with non-spreadsheet data. BioPython is a library for working with general sequence datasets at a variety of scales ( Cock *et al.,* 2009). More specialized libraries, such as Poretools ( Loman & Quinlan, 2014) (for Nanopore data) or Dendropy ( Sukumaran & Holder, 2010) and ETE3 ( Huerta-Cepas *et al.,* 2016) (for phylogenetic trees), are also available for work in subdisciplines of biology and bioinformatics.

Depending on the aims of the class, it may be necessary to choose a supporting language, as well. For example, in contrast to a coding-to-learn course in which biology students practice drawing conclusions from data, a course on genomics might require students to interact with large datasets on a remote server. Many such servers require a UNIX command line to access. While this article will focus on R and Python, some instructors may find that they need additional languages to support student learning, particularly in advanced disciplines. Furthermore, while we suggest focusing on a single language for pedagogical reasons, and highlight R and Python, our goals in choosing a language are (1) to select one that is widely used with available tools, and (2) has straightforward syntax for novices ( Stefik & Siebert, 2013). We support the philosophy taken by the Software Carpentry organization of using any language that meets these goals and provides a platform for learning the fundamental process of automating data analysis.

## Local computing platforms

### Local installs on personal computers

Perhaps the most basic computing platform setup is to have learners install languages and software locally on their own computers. Local installs may work best in small classes that focus on the basics of coding, where the goal is for learners with no prior experience to leave the course self-sufficient to do computational work. R is easily downloaded and installed from the R-Project website. RStudio provides a commonly-used integrated development environment (IDE) that allows management of additional packages and visualization of data and figures. The Anaconda installation of Python provides the programming language, as well as the Jupyter notebook environment for simultaneously viewing code and output, and the ability to run code in blocks. The Anaconda installation also provides the conda package manager for installation of additional packages.

***Advantages of local installs.*** Local installs allow learners to immediately be able to apply their new knowledge of code outside the class without additional support. For learners who are already familiar with their own computers and operating systems, having students use local installs can bypass some of the learning curve associated with learning a completely different platform; this also applies for instructors, who often have limited time to devote to learning a new platform exclusively for the purpose of teaching a course.

If the software used in class does not require a paid license or subscription, learners will continue to have access to the specific tools used in class after the course ends. Teaching learners with tools that they know they will be able to take away with them and apply in the future increases learner motivation to learn these tools, and increases the chance that they will actually use them in future work ( Kember *et al.,* 2008).

Local installs are a solution in particular for smaller institutions that do not have access to remote computing platforms. In some cases, though setting up a computing platform may be theoretically possible, it may not be feasible within the constraints of resources, time, and IT personnel. Local installs offer an alternative system that does not typically require extensive coordination with IT personnel at an institution. Additionally, local installations avoid the challenges presented by a poor internet connection and dependency on a single outside computing platform.

***Challenges of local installs and how to overcome them.*** While using local installs can minimize time spent learning new platforms, it may also extend the initial time spent setting up learners’ computers for the course – namely, with language and software installation. The feasibility of using local installs may thus be limited in many cases by class size. This up-front time can be minimized by having learners complete as much installation as possible before the course begins using detailed instructions or videos available on the software developers’ websites or provided by the instructor. For example, Data Carpentry provides installation instructions as part of its workshops and the University of British Columbia provides installation information to support its Data Science courses. However, some guidance may be necessary either during class or individually. In addition, troubleshooting installs on various platforms can be challenging, especially with a larger class working on a wider variety of computers. In some cases, software may not be available for a particular platform (e.g. RStudio on Chromebooks). Further, if learners have previously installed a particular language, IDE, or package, they may have a different version than that used in class, which may complicate analyses later. This issue would present less of a problem in classes that primarily cover the foundations of coding, and/or with learners who have little to no prior experience.

In classes that have learners do analytical work requiring substantial computational power, learners may not have computers that are able to do that work. Some learners may not have computers at all. For these reasons, local installs may not be the best option for a required course (i.e., if a learner must take the course regardless of whether or not they have a computer they can bring to class) or for a course where a substantial amount of time is spent on computationally intensive work. For elective courses, the instructor may choose to list a computer as required material for the course and make that information available to students during the class registration period. However, this requirement limits the learners who are able to take the class to those who have a computer to bring. With small classes, it may be possible to provide a computer to a learner who does not have their own, and the instructor should also ensure that the learner has access to a computer outside the classroom with necessary software for homework. An alternative solution may be to hold the class in a computer lab, but installation of software in computer labs may require IT support, depending on the institution.

### Single-board computers (SBCs)

Single-board computers (SBCs) are minimalist, complete computers built on a single circuit board. There are a number of organizations in the single-board computer market, but they were first popularized by the Raspberry Pi Foundation. The most basic SBC models cost as little as $10 and offer WiFi, Bluetooth, and quite high-performance computing. Generally, a Linux distribution, such as Raspbian, is installed on a microSD card and run on these machines.

An SBC is not immediately useful on its own, because it does not have a monitor or input devices. However, these machines can be used (1) as a server, by logging in “remotely” from another machine via WiFi or wired connection — for example, using secure shell (SSH) protocol — or (2) as a standard Linux PC, by booting into the desktop environment and using attached input and output devices (e.g., monitor, keyboard, and mouse). Having learners use SBCs shares some similarities to using local installs, in the sense that the learners can run software installed on their own local machine. However, SBCs would ensure that all learners are using the same computing environment and have the same capabilities. Furthermore, SBCs can be used together with all the other computing platforms we discuss here.

***Advantages of SBCs.*** Teaching with SBCs can be particularly effective at an institution with limited IT support. Even in a classroom without reliable internet, the instructor can set up an SBC as a local network for sharing code and data among the learners. Thus, they can also be used in teaching environments not traditionally thought of as computing-friendly, such as in the field.

SBCs can be particularly good tools for teaching learners how to use remote Linux servers and HPC resources. Remote computing can be an abstract concept for students with limited computational background. Showing students how to “remotely” login from another computer to their SBC they have in-hand can make the idea of remote computing much more tangible. Furthermore, SBCs offer a way of teaching high-performance computing when such resources are not available. Even institutions with HPC facilities often do not support the use of these resources for teaching. On a very modest budget, an instructor can set up a shared, “mini” computing cluster to teach fundamental principles of HPC, such parallelization, message passing, and scalability.

One particularly exciting pedagogical opportunity presented by SBCs is the integration of tech and computation to create biology curricula needed by students in the 21st century (see Box 1). Imagine a scenario where all learners entering a program would receive an SBC along with some basic training on how to use it. Because SBCs allow direct access to the computing board and associated headers and ports, they are extremely modular, and can be coupled with many inexpensive accessories for collecting data. For example, for as little as $40, one can purchase an add-on board that includes a variety of sensors, including a gyroscope, accelerometer, magnetometer, temperature, barometric pressure, and humidity. Learners using these devices could collect observational or experimental data in the lab of their introductory biology classroom. Then, in a lecture or discussion session, they could learn to use basic scripting to process, visualize, “clean,” and analyze those data. If an undergraduate or graduate curriculum was designed for the students to continue using their SBC, data, and scripts throughout their coursework, this could be a powerful approach to training the next-generation of computationally competent biologists. The development of such a curriculum goes far beyond the scope of what a typical teacher needs from a computing platform. Nonetheless, this aspirational idea is worth highlighting when discussing SBCs, because they can be used jointly with all the other computing platforms discussed in this paper.

Box 1. **Diversity and Inclusion Callout Box 1**

The low cost of high-performance, single-board computers (SBCs) can make the classroom more equitable. With a cost as low as $10 per computer, it requires minimal investment from learners, or, more ideally, increases the likelihood that an institution or funding agency can cover this cost. Using SBCs also ensures that all learners experience the same computing environment and capabilities. Furthermore, the low cost can allow the learners to keep the computer after the course is over. This can enable life-long learners across all socioeconomic backgrounds.

***Disadvantages of SBCs.*** The initial setup of an SBC requires time and effort to install the Linux distribution on the microSD card. If the primary goal of the course is to use technology to teach biology, this work should be done by the instructor prior to the class. If the class is large, this would most likely require IT support to pre-install the operating system on many microSD cards. However, if any of the learning objectives of the course involve a better understanding of operating systems or computer hardware, turning the initial setup into an active-learning exercise done at the beginning of the class could be a very informative learning experience for the students.

Even after the initial setup, SBCs are not immediately useful on their own. Learners require either another computer to login to the SBC via SSH, or peripherals (e.g., monitor, keyboard, and mouse) to use the SBC as a PC. By the time these peripherals are factored in, the total cost could become similar to an entry level laptop. However, the learners can benefit from the modularity of the SBC system. The peripherals can be re-used, and the students could keep the SBC itself. This is not possible with laptops.

When not used as a PC (i.e., without peripherals), using an SBC can be quite unintuitive for learners not familiar with the Linux command line. For example, it would be counter-productive to take the time necessary out of an introductory biology class to teach learners to become proficient in using an SBC from the command line. While this weakness can be overcome by using the SBC as a PC, this option does require additional investment and logistics to provide the learners access to keyboards, mice, and monitor.

## Remote computing platforms

A number of platforms for serving course materials via the internet have become available. Because these technologies share many similarities, we will first discuss these computing platforms generally, then discuss teaching with R (such as with RStudio server, per RStudio Team (2015)) and Python (such as with JupyterHub, per Kluyver *et al.* (2016)) in the cloud. Remote computing platforms may be used by purchasing time on a commercial server (often colloquially called “the cloud”), or can be deployed on machines owned by the instructor or the university (such as a server or high-performance cluster computing unit).

### Advantages of remote computing platforms

Remote systems typically provide a web interface to a centralized version of an IDE. This allows the instructor to set up a uniform instance for all learners, avoiding time spent sorting out problems, such as learners having different versions of languages or software installed. Learners can then focus on learning biology and/or working with data, rather than on managing technical details.

Remote computing platforms also ensure that learners are not limited by their hardware or software. The instructor sets up the class software on a server, rather than the learners installing software on their own personal machines, thereby avoiding problems such as learners not having computers powerful enough to run exercises, or computers without the ability to do language installs (such as NetBooks). This can be especially important for serving students who have difficulty purchasing a personal laptop to bring to class (see Box 2).

Box 2. **Diversity and Inclusion Callout Box 2**

A local RStudio Server or JupyterHub reduces barriers to the use of R or Python and support underprivileged students by removing the requirement to have a laptop (a Chromebook or tablet is sufficient) and minimizes the frustration associated with installation and different operating systems. Students can access the server from any location via a web browser using any machine while experiencing a consistent interface and access to their code and data.

Many remote computing platforms are agnostic to the type of machine on which they are run. For example, JupyterHub and RStudio Server can be deployed on any machine with Unix or Linux supported ( RStudio Team, 2019). This machine can be owned by an instructor, a high-performance cluster (HPC) computer operated by a university, or cloud computing resources rented by the instructor. The main considerations for choosing what type of machine to run a remote computing platform are memory, storage, and access. For courses with low-memory tasks, such as data processing on small datasets, a single machine may be sufficient, even with large numbers of users. For high-memory tasks (such as genome assembly), or tasks that with long runtimes (such as phylogenetic estimation), cloud compute resources that can be resized from week to week may be more suitable. For example, platforms such as Digital Ocean and Amazon Web Services, allow users to pay for more memory only when needed. The computing instance can be resized between class periods to allow more memory to be available to learners. This flexibility can allow an instructor to run a course on the resources they have available to them, based on their available infrastructure and funds.

If faculty are deploying the course on a local server, such as a personal server or a university-owned HPC unit, they may require IT support to set up the server, especially if the course is large. However, in our experience once setup is complete, maintenance requires minimal time and having IT handle upgrades and additional users relieves faculty of some work. The size of the server required for even large numbers of students to do analyses can be quite minimal (e.g. just 4 cores for hundreds of students), provided they are conducting analyses of relatively small datasets (e.g. hundreds of samples).

### Disadvantages of remote computing platforms

While the remote computing platforms are a flexible tool for teaching, there are some downsides. Because students typically interact with the server via a web-based login, if university resources are to be used, instructional technology (IT) staff will typically want to be involved. Some universities do not allow public-facing servers to be operated by non-IT staff, which can limit instructors from using computers they have on hand to serve their coursework. Likewise, due to security concerns, it may not be possible to operate a server on a university HPC. In this case, the instructor will likely have to turn to cloud compute providers. Many of these providers are affordable for small classes or low-memory tasks, but costs will scale with users, storage, and memory requirements.

These technologies do interact with the internet. Therefore, when problems arise in the classroom, the instructor must distinguish between problems with the server itself, with a learner’s computer, and with other classroom technology, such as wireless internet. While internet-based technologies can increase for equity and inclusion by allowing learners to interact with the course materials no matter how old their computer is, or if learners have unreliable computer access, they increase the complexity of diagnosing performance problems. Unstable internet connections can cause many, if not all, members of the class to lose connection to the materials during class. Learners with individual computer problems, such as malfunctioning wireless cards, may need to borrow a computer to access materials. If the instructor is teaching without IT support, they may need to halt class and fix problems if the server itself is malfunctioning.

Additionally, because a server facilitates learners focusing on the data and analyses, rather than installation and troubleshooting, learners may leave class without the ability to work independently outside the classroom. They have not learned how to install the programming language or IDE, and these are not installed on their personal computer. Additionally, they do not have or know how to install supporting packages. If the goal is for students to be equipped for independent work, the instructor should be sure to introduce these skills explicitly during the semester.

### RStudio server

RStudio Server ( RStudio Team, 2019) provides a web interface to a centralized version of the RStudio IDE for working with R code. By using RStudio learners are able to simultaneously view their script, environment variables, file structure, plots, installed packages, etc. The Server format allows the administrator to maintain a consistent version of R, RStudio, and installed packages. This approach allows beginning coders to focus on data and results, rather than worrying about installation and versions. It allows an instructor to be sure that learners can focus on their code without being limited by their hardware or software.

Some advantages of RStudio Server are that learners’ data and code are stored (and backed up) on the server. Instructors can also access a student’s code from anywhere to help students solve problems remotely. RStudio Server is free for teaching purposes upon submission of a syllabus to the RStudio Company. Additionally, for smaller scale teaching an instructor may use RStudio Cloud, which provides a free ready-to-go web-based version of RStudio.

It should be noted that RStudio can accommodate other languages, mostly through the use of external packages. For example, the R package Reticulate provides support for Python ( Ushey *et al.*, 2020), and the package Knitr ( Xie, 2014; Xie, 2015; Xie, 2020) allows the use of various scientific computing languages. Furthermore, other remote computing platforms, like Jupyter, support the R language.

### JupyterHub

Project Jupyter is a non-profit organization that creates open-source tools for computation. One of their most famous products is the Jupyter Notebook, which allows researchers to create interactive code documents. These documents can have text, images, and code cells that run and render output for inspection. The Jupyter Notebook was originally developed as the iPython notebook, but has since expanded to include language support for other popular languages such as R and Julia. A JupyterHub is a server on which an instructor can deliver lessons via Jupyter Notebooks to a full classroom of students using a standardized server environment. In most cases, the server will be accessed via the internet. The first and foremost reason to use a JupyterHub is that you want to teach with the Jupyter ecosystem of tools. The Jupyter Notebook is commonly used in education in the sciences. It allows the instructor to develop a set of instructional materials that include text, code, and images. In this way, detailed notes can be provided from the instructor to the learner, and the learner can augment these lessons with their own notes, annotations, and code. Though originally invented for use with Python, Jupyter Notebooks are compatible with a number of languages. Multiple languages can even be used in one notebook, making them ideal for use with multi-language pipelines. The JupyterHub also has a command-line terminal and the Git version control system installed, for more advanced courses.

Jupyter supports the R language, and using Jupyter Notebooks with R can be a powerful teaching tool. However, JuptyerHub does not support popular integrated development environments (IDEs), such as RStudio or Python’s Spyder. While there is a text editor for preparing scripts, and a command line to run scripts, the JupyterHub is really designed to serve Jupyter Notebooks, and the interface caters to this technology.

### HPC and cloud

RStudio Server and JupyterHub will typically require that instances are *hosted* somewhere. This refers to having the compute platform installed in a central location, at which it can be accessed by all students. It may be possible for an instructor to host their course platform on a computer or server they personally own. However, in the experience of the authors, many universities prohibit faculty from hosting servers on-campus that will be available off-campus due to security concerns with allowing off-campus computers to access on-campus resources. This means that you may be able to host a server via the intranet, but if students go home for the weekend, or live off-campus, they’ll need to come back on to campus to do their homework.

These limitations cause many instructors to look for alternative ways to provide their course platforms to students. One common way is by working with a local high-performance cluster compute facility. A high-performance cluster computer is a set of computers that are networked. This allows researchers to harness the power of several computers at once. These facilities often also service educators. A benefit to serving course content via high-performance cluster computing is that these resources are often free for educators at the university where the computer resides. Because these services are often in-house, the administrators of them are able to handle any specialized security concerns with the course platform. National level high-performance cluster computers, such as CIPRES ( Miller *et al.,* 2010), JetStream ( Stewart *et al.,* 2015; Towns *et al.,* 2014) and CyVerse ( Goff *et al.,* 2011; Merchant *et al.,* 2016) also offer resources for US institutions. For campuses with limitations on HPC use, IT may be able to set up an independent virtual machine on other existing university servers.

However, not every university has a high-performance cluster computer that is set up to host coursework. In this case, an instructor may want to consider a remote solution. The concept of a remote solution is similar to using high-performance cluster computing. An educator can rent one or more computers to serve their coursework. Cloud computing can take several forms. In some cases, the instructor explicitly rents computers in sizes that are appropriate for their course. These types of services often charge by the number of computers, the size of the hard drive rented, the amount of memory on the computer, and the amount of time for which they will run. Examples of this type of service include Digital Ocean, Cloud Flare, Google Cloud, and Amazon Web Services. However, it is important to note that if you are signing a contract for cloud services, it is prudent to check with your IT, legal, and purchasing departments. Departments may have rules in place for student privacy and data security, as well as for contract bids between providers.

Recently, educational cloud service providers have entered the market. Examples of these services include RStudio Cloud and Python Anywhere. These types of products are oriented towards providing an environment that is oriented towards a specific language or course platform. For example, RStudio Cloud hosts RStudio instances, and Python Anywhere hosts a variety of Python environments, including Jupyter notebooks. Like an regular cloud provider, these services typically charge based on the size of the amount of memory and storage capacity of the computer needed. Being oriented towards education, some of these providers also offer accounts for free, with paid accounts available for users who need more memory or computer power.

### Bridging the gap between local and remote approaches

Container software, such as Docker, Singularity, or Podman, offer a way to teach with the simplicity of local installs while utilizing the consistency of remote computing platforms. Containers allow the instructor to create a standard computing environment that each student can work in on their own computer. Instead of having to install multiple tools that will be used in the course, the students would only have to install the container software (e.g., Docker, which is cross-platform), and then they can download and run the environment created by the instructor. The functionality is very similar to a virtual machine, but lighter-weight and more efficient. Containers can be easily updated and distributed with remote services like DockerHub. This allows the instructor to update the students’ computing environment in real-time. Of course, this still has the same disadvantages discussed above of having students run analyses on their own computer.

## Teaching methods

Designing an effective biology course that incorporates computing requires more than choosing the best computing platform for your needs. Skills-based courses such as coding necessitate a unique approach to teaching that can differ in some ways from more commonly used pedagogy in content-based courses. For many learners, a biology course that involves hands-on computing may be their first classroom experience that heavily involves learning increasingly complex skills that build off of each other, rather than primarily learning content. And similarly, instructors whose research and teaching expertise is in a field that is not strictly computational — which likely includes most instructors of biology — may benefit from guidance on best practices for delivering content effectively and addressing the unique challenges that arise. As a skills-based discipline, computation necessarily requires some level of active in-class interaction from learners (with the instructor or peers, or with the material itself). Thoughtful implementation of appropriate active learning strategies has been shown to increase both mastery of content and development of general skills such as critical thinking and collaboration ( Faust & Paulson, 1998). Below, we describe several strategies for teaching coding, and address challenges that instructors may face in teaching a course that is perhaps more similar methodologically to math and foreign language than to other biology courses. We acknowledge that each section summarizes and simplifies a significant body of literature and encourage readers to pursue a deeper understanding of pedagogy elsewhere. For example, the recent book “Teaching Tech Together” ( Wilson, 2018), offers a concise overview of many of these issues.

### Example-based learning

Example-based learning has a long history in teaching coding, and consists of the instructor providing examples of how to solve a given problem using code. Sometimes, these examples are static and provided in a textbook or on slides. Other forms of example-based learning require the learners to actively engage with the code. An especially effective way to teach computational biology is to combine multiple forms of example-based learning ( Renkl, 2014a). For example, an instructor might use all three methods detailed below: first show learners some written examples, perhaps in pre-reading for a class, then use live coding during class and have the learners follow along, and subsequently have learners complete a series of examples with increasing amounts of code that learners need to fill in during in-class problem sets or in homework assignments.

***Written examples.*** Written examples are often a good starting point to introduce learners to the concepts and structure of solutions. Written tutorials allow learners to go at their own pace. This ensures learners take the time they need. However, learners frequently skim over static written examples or skip formative assessment questions, confident that they understand what the code is doing. Students may need to be required to critically evaluate the code to truly ensuring their understanding. For this reason, written examples are most effective when integrated with other example-based learning methods ( Renkl, 2014a).

***Live coding.*** Live coding is a form of example-based learning in which learners watch the instructor solve problems and in most cases the learners follow along. The act of typing along forces learners to actively enter code, which is a hands-on version of a worked example. This means the instructor must proceed at a reasonable pace that learners can follow. The instructor can stop and ask learners challenge questions as formative assessment to ensure that learners apply their knowledge immediately and question their own understanding. The instructor can also set up a problem, and pause to have learners figure out the rest of the exercise. This live coding approach has been popularized by Data and Software Carpentry and similar coding workshops. Live coding can help teach aspects of programming that are not easily taught by viewing static code, such as incremental coding (writing a few lines and testing them) and debugging. From the perspective of the learners, live coding is generally preferable to viewing static code, especially when learners are able to code along with instructors ( Raj *et al.,* 2018).

A disadvantage of live coding is that everyone goes at the same pace. This may be too fast for some and too slow for others. One possibility to alleviate this disadvantage is to flip the classroom and provide learners with pre-recorded live-coding experiences. The learners then come to class where they complete an exercise or a series of faded examples.

***Faded examples.*** Faded examples are a form of example-based learning that can be more effective than static worked examples ( Schwonke *et al.,* 2009). Faded examples include sets of worked examples, beginning with a complete example. With each subsequent example, key problem solving steps are removed and learners must insert the steps themselves ( Renkl, 2014b). Faded examples have been demonstrated to require less learning time with potentially better learning outcomes ( Schwonke *et al.,* 2009).

In the context of teaching computation to biologists, faded examples are particularly facilitated by servers. For example, a Jupyter notebook containing faded examples may be made available to learners on the server. If learners successfully complete the code they will produce an expected outcome, thus allowing for the learner to self-correct their work. Fillable Jupyter notebooks using the faded examples technique force learners to engage with the material and actively problem-solve. Fillable notebooks are particularly useful because they run the code and allow the learner to immediately discover if they produced the right answer. This type of immediate feedback allows the learner to work towards the correct answer, rather than turning in work that they believe to be correct and receiving a low grade on something they put time and effort into. An important component is providing learners with a solution set after they have had the opportunity to solve the faded examples so that they can see where they went wrong if they are unsuccessful.

***Additional example-based exercises.*** A variety of exercises are frequently used in testing and reinforcing learning of computational concepts that do not require learners to create new code. For example, learners could be presented with code that contains a bug and be tasked with providing the Minimal Fix that would allow the code to run. Alternatively, the learners could be presented with a series of lines of code and be asked to rearrange them such that they successfully complete a task (“Parsons Problems”; Parsons & Haden, 2006), or to trace the order in which code is executed (for instance, within a series of nested loops or if statements). See the section “Exercise Types” in Wilson (2018) for more detail and exercises of this variety.

### Focusing on coding as problem-solving

One of the main skills underlying learning to code is learning how to solve problems. Writing code can be viewed as a method of communicating to the computer the precise steps of the analysis. In fact, code serves as a written record of the analytical steps, and is one that is instantly reproducible. When the learning outcomes are not necessarily tied to learners having full mastery of a specific language’s syntax, shorthand notation can be used to outline the steps the learner would take to get to the end result, removing the added layer of correct syntax and highlighting the importance of the problem-solving elements. This method, called pseudocode, can help learners logically scaffold their thought process, and therefore their code, regardless of which language they will be coding in. Learners can be required to write the solution to their problem in pseudocode to demonstrate their thought process, and then use that pseudocode to write their code (e.g., Olsen, 2005). Courses may rely on pseudocode during timed exams to relieve exam stress. Note, however, that if learners are expected to interpret or elaborate on pseudocode — especially in an exam setting — that the meaning behind each pseudocode term is clearly and unambiguously defined ( Cutts *et al.,* 2014).

### Cooperative learning

Working in groups or pairs can result in numerous positive outcomes in the biological sciences, including enhancing scientific thinking and attitudes about biology, aiding in the instruction and evaluation of course material, providing a better understanding of practical skills and their applicability in real-world environments, improving reading, writing, and social skills, and supporting learning for a broader array of learners (reviewed in Lord, 2001). In the context of teaching computational biology, group projects and other forms of cooperative learning have frequently been used to facilitate example-based learning (e.g., Emery & Morgan, 2017; Fuselier *et al.,* 2011; Korcsmaros *et al.,* 2013). One cooperative learning teaching tool for computational biology is pairing learners up and having one student be the ‘driver’, who types in the code, and one be the ‘navigator’, who has to tell the driver what to type. This exercise forces learners to think carefully about the process of creating code. Even if learners are not expected to work through examples or produce a project in pairs or groups, cooperative learning can still be implemented by facilitating discussion and collaboration among learners. This can be done in class (e.g., through think-pair-share exercises, in which learners are encouraged to think about a problem individually, then discuss in pairs or small groups) or outside of class through discussion forums. For instance, learners could be encouraged or required to participate in discussions on the course website (e.g., Blackboard/Moodle) or a Slack workspace created for the class, or to do peer review of other learners’ projects. Learning from each other can be a powerful way to get learners engaged with the course work, and can solidify their understanding by having them teach each other ( Treisman, 1992).

However, as most students and instructors know, group work can create problems and strife. Interpersonal issues within pairs or groups can hinder the learning process. Sometimes groups or pairs are unbalanced with regard to prior knowledge, speed of learning, or effort contributed to the project. Additionally, group size or composition can lead to an entire group struggling to succeed (e.g., Compeau, 2019). Therefore, although group projects are frequently used in computational biology and can be a useful tool, relying only on group-based projects for critical assessments in a course may not be the best solution.

### Other challenges and tools

Incorporating computational biology into the classroom comes with additional challenges that may not be present in other biology courses. Many learners will have some prior biological knowledge but have no experience with computer coding. This creates a situation where learners have many questions but often feel out of their comfort zone, or are simply uninterested in the computational components of the course material. These factors combine result in a number of different challenges for instructors to help learners successfully navigate the computational side of a biology course.

***Demonstrating value.*** The unfamiliarity of coding can sometimes lead learners to push back against the idea of learning to code. In our experience, demonstrating how coding can be a useful tool for doing the same analysis multiple times (e.g., generate the same type of plot for different datasets) helps learners see the value in learning to code. This demonstration is especially powerful when the learners are enabled to discover the utility of coding on their own. For example, an instructor could provide students with a large dataset and encourage the learners to try basic analyses (e.g., producing summary statistics or basic plots) in both R and Excel. Particularly when learners have not opted into learning computational skills — for example, when computational skills are incorporated into required biology classes — showing learners the value of saving future time and effort is very important.

***Teaching how to ask for help.*** Not all learners will automatically ask for help (see Box 3). As instructors, we need to encourage learners to seek our help when they are struggling. One method we have found successful is to provide learners with rewards for asking for help, such as an automatic extension on deadlines if learners attend office hours. Having knowledgeable teaching assistants or support staff to circulate the classroom and check on learner progress during in-class exercises is also helpful (see Box 3 for further notes on asking for help). In these cases, instructing the teaching support staff to first approach learners who do not have their hands raised will provide help to a larger proportion of the class, especially many learners who need the most help. To help learners signal that they need help, instructors can use a system that does not force students to stand out by raising their hands. For instance, Data and Software Carpentry use post-it notes to discreetly signal to instructors when learners need assistance. Incorporating live coding into lectures is another way to normalize asking for help; while live coding, instructors can demonstrate that errors are a natural part of coding, how to effectively search for solutions to problems, and how to look up syntax details.

Box 3. **Diversity and Inclusion Callout Box 3**

Asking for help is critical for learners to be able to overcome confusion, solidify their skills, and deepen their knowledge and understanding. However, cultural differences exist that may prevent learners from seeking help, even when they need it most. Universities in particular have the unspoken expectation that undergraduates will seek help when they need it, but people from some backgrounds will not necessarily know this, or may feel uncomfortable approaching their teachers, who are in positions of authority ( Jack, 2016). Different cultures may perceive asking questions in class in different ways; some cultures view questions as a way to gain recognition, whereas others view questions as a demonstration of ignorance. The latter group are less likely to ask questions in class and thus do not receive either needed help or engagement with the instructor.

***Large class sizes.*** For instructors who are looking to incorporate computational biology into large classes, such as in an introductory biology course, the prospect of testing and grading code turned in as assignments may be daunting. We have found that relying on knowledgeable TAs, support staff, and demonstrators is the best way to ensure that all learners receive the help that they need. Placing learners in pairs or small working groups (see the section on Cooperative Learning above) can also provide learners with support and feedback that will facilitate their learning, without placing the entire burden on instructors and TAs. Additionally, relying on IT support to help manage the computing platforms can reduce the workload for the instructors. Furthermore, using assignments that can be automatically graded (e.g., mimir classroom, nbgrader in Jupyter, or OK) will help reduce the time teachers must spend grading. An added benefit of such autograding technologies is that they can provide students with immediate feedback on their performance and understanding of the computational materials.

***Plagiarism.*** Writing code is more similar to math than to writing, as there is a correct answer (or perhaps several correct answers), and it can be difficult to detect whether learners arrived at that answer independently or through copying someone else’s solution. Plagiarism can be exacerbated by the common phenomenon of searching for solutions to a given problem online and copying or adapting posted solutions without attributing proper credit ( Gaspar & Langevin, 2007). Some of the programs that grade code include plagiarism detection components ( Pears *et al.,* 2007), which can help instructors identify when plagiarism has occurred. One approach to ensure learners understand code is to require commented explanation of each component of their code, and ensure these comments are different for each learner. Requiring unique comments from learners has the advantage that it allows learners to be able to search for the functions and arguments to functions that are needed to implement their analytical work, which is common practice when writing code. Another approach is to allow learners to work on assessments as a group, but have each learners write out their own pseudocode explanations of the code. Instructors are encouraged to discuss issues surrounding intellectual property and plagiarism and reinforce the importance of citing sources.

## Conclusions

Computing is increasingly being incorporated into coursework at all levels in biology. Computing is both a key skill for learners and supports additional learning by enabling the incorporation of data and analysis into classrooms. Instructors have a range of choices to deliver computational lessons. From the computing platform to how the instructor delivers the lesson, every aspect of a computational classroom can shape learners’ knowledge and confidence. In this paper, we have outlined the costs and benefits of several popular computing platforms. Given the variety of options, instructors should be able to find a platform that best fits their course learning objectives, available resources, class size, and learner backgrounds. One important consideration in this decision is how the platform of choice affects learner access to course content. Many of these computing platforms can support inclusion in the classroom (and thus, ultimately, in fields and careers that incorporate computation) by providing access to the same technologies to all learners. We have also discussed several teaching strategies for computational biology and other courses with computation, with the goal of improving student engagement, learning, and retention.

## Data availability

### Underlying data

There are no data associated with this article
