# Supplementary material for: The why, when, and how of computing in biology classrooms
Source: F1000Res. 2020 Mar 2;8:1854. Originally published 2019 Nov 5. [Version 2] doi: 10.12688/f1000research.20873.2 (PMC6971840; doi:10.12688/f1000research.20873.2)
Supplement: Peer review history

# Peer review: Reviewer response for version 1

**DOI:** 10.5256/f1000research.22961.r57707

**Published:** 2020-01-20

**Authors:** Thomas Kluyver

**Affiliations:** 1 European XFEL, Schenefeld, Germany

The article gives an overview of different available options and considerations for adding a programming component to a university-level biology syllabus. The authors don't give a one-size-fits-all answer, choosing quite reasonably to highlight where different tools make most sense. I appreciated the callouts highlighting issues that could affect some students more than others.

 In the context of teaching with Jupyter notebooks, I would mention nbgrader, an open source tool which works with JupyterHub to support assignments in notebooks, with semi-automated grading.

 I think the article could be made clearer by using the word 'cloud' more carefully. Admittedly marketing has made the term extremely nebulous in general, but it's used here with two distinct meanings: first for providing software on a central server or cluster, and then more specifically for commercial services which rent computing resources.

I confirm that I have read this submission and believe that I have an appropriate level of expertise to confirm that it is of an acceptable scientific standard.

# Peer review: Reviewer response for version 1

**DOI:** 10.5256/f1000research.22961.r56230

**Published:** 2019-11-26

**Authors:** Tiffany Timbers

**Affiliations:** 1 University of British Columbia, Vancouver, BC, Canada

The authors provide a nice introductory overview of teaching computational biology. They cover several key practical and pedagogical aspects one needs to consider when designing and teaching a computational biology course. These include choice of programming language, choice of computing platform, and best practices for teaching computational materials. This review will serve as a good overview of the field at the present time for newcomers. 

 Below I suggest some things that could improve the manuscript:

 In the introduction, where the rationale for teaching computation in biology classes is explained. I suggest adding an additional rationale: to give students the tools, skills and motivation to create analyses that are reproducible and transparent, regardless of the size of the data set ("it's worth it even for small data sets).

 I agree that Software and Data Carpentry workshops are great places to start to get computational skills, however to learn the skills to teach computation to others, faculty will need additional training. For example the Software and Data Carpentry Instructor training courses (perhaps this is what you were referring to?). Another resource that is aligned with this is " Teaching Tech Together" by Greg Wilson.

 In the "Choice of computing language" section, the two paragraphs comparing R and Python could be improved. For example, both R and Python now have easy to use tools for working with rectangular data, but that is not made clear in the Python paragraph. The authors do write that "Pandas can be used for teaching foundational data skills", but someone unfamiliar with Pandas will not likely make the leap that manipulating rectangular data is what is meant here. Essentially, for simple, rectangular data tasks, that do not involve complex statistics, both R and Python are excellent language choices. And one should pick the one that their colleagues and collaborators use. However, when analysis tasks get more specific, then one might have to use the language where the package exists with specific tools they need. 

 In the first paragraph of the "Cloud-based computing platforms" section, I think it is important to communicate that R (and other languages) can be used with Jupyter. This is stated later, but R readers might skip the Jupyter section later on if this is not communicated earlier. Also, I would remove the sentence "Jupyter supports the R language. However, the interface for R in Jupyter is different than RStudio, a dominant way to interact with R". Jupyter is getting better and better at working with R, and thus if only a notebook is needed, there is no reason not to use Jupyter with R. However, if your code project is more complex and needs/benefits from a real IDE, then of course RStudio should be used (and the same argument goes for Python).

 In the "Teaching methods" section attribution is given to Data Carpentry for popularizing the live coding approach of teaching coding. However, Software Carpentry proceeded this. This also happens again later in the manuscript when the post-it note system is discussed. In earlier parts of this manuscript, Software Carpentry is referred to alone. Perhaps it would be better to refer to these two organizations together consistently throughout the manuscript as the "Software and Data Carpentry organizations" (or something like that). 

 There are several other great coding exercise strategies that would be great to point out in this article (Parson's problems, tracing (execution and values), minimal fix, refactoring, etc). These are described quite well in " Teaching Tech Together" by Greg Wilson.

 One last addition I might add to the platform and/or teaching method section is the potential for the use of autograding and/or automated feedback using tests in computational biology. When giving students a computing challenge, tests can also be provided to see if the student's code creates an object or function with the desired properties or functionality. In Jupyter notebooks, these tests can be paired with tools such as nbgrader or OK for autograding. This is being used in several undergraduate Data Science courses at several universities (e.g., Berkley & the University of British Columbia, etc).

I confirm that I have read this submission and believe that I have an appropriate level of expertise to confirm that it is of an acceptable scientific standard.
